# Evaluation of *in vitro* cytotoxicity, biocompatibility, and changes in the expression of apoptosis regulatory proteins induced by cerium oxide nanocrystals

**DOI:** 10.1080/14686996.2017.1319731

**Published:** 2017-05-31

**Authors:** Shahanavaj Khan, Anees A. Ansari, Christian Rolfo, Andreia Coelho, Maha Abdulla, Khayal Al-Khayal, Rehan Ahmad

**Affiliations:** ^a^ Nanomedicine & Biotechnology Research Unit, Department of Pharmaceutics, College of Pharmacy, King Saud University, Riyadh, Saudi Arabia; ^b^ Department of Bioscience, Shri Ram Group of College (SRGC), Muzaffarnagar, India; ^c^ King Abdullah Institute for Nanotechnology, King Saud University, Riyadh, Saudi Arabia; ^d^ Phase I- Early Clinical Trials Unit, Oncology Department and Multidisciplinary Oncology Center Antwerp (MOCA), Antwerp University Hospital, Edegem, Belgium; ^e^ Colorectal Research Center, College of Medicine King Saud University, Riyadh, Saudi Arabia

**Keywords:** CeO_2_-nanocrystals, nanotechnology, *in vitro*, cancerous cell lines, apoptotic pathways, hemolysis assay, 10 Engineering and Structural materials, 102 Porous / Nanoporous / Nanostructured materials

## Abstract

Cerium oxide nanocrystals (CeO_2_-NCs) exhibit superoxide dismutase and catalase mimetic activities. Based on these catalytic activities, CeO_2_-NCs have been suggested to have the potential to treat various diseases. The crystalline size of these materials is an important factor that influences the performance of CeO_2_-NCs. Previous reports have shown that several metal-based nanocrystals, including CeO_2_-NCs, can induce cytotoxicity in cancer cells. However, the underlying mechanisms have remained unclear. To characterize the anticancer activities of CeO_2_-NCs, several assays related to the mechanism of cytotoxicity and induction of apoptosis has been performed. Here, we have carried out a systematic study to characterize CeO_2_-NCs phase purity (X-ray diffraction), morphology (electron microscopy), and optical features (optical absorption, Raman scattering, and photoluminescence) to better establish their potential as anticancer drugs. Our study revealed anticancer effects of CeO_2_-NCs in HT29 and SW620 colorectal cancer cell lines with half-maximal inhibitory concentration (IC_50_) values of 2.26 and 121.18 μg ml^–1^, respectively. Reductions in cell viability indicated the cytotoxic potential of CeO_2_-NCs in HT29 cells based on inverted and florescence microscopy assessments. The mechanism of cytotoxicity confirmed by estimating possible changes in the expression levels of Bcl2, BclxL, Bax, PARP, cytochrome c, and β-actin (control) proteins in HT29 cells. Down-regulation of Bcl2 and BclxL and up-regulation of Bax, PARP, and cytochrome c proteins suggested the significant involvement of CeO_2_-NCs exposure in the induction of apoptosis. Furthermore, biocompatibility assay showed minimum effect of CeO_2_-NCs on human red blood cells.

## Graphical abstract

**Figure:**
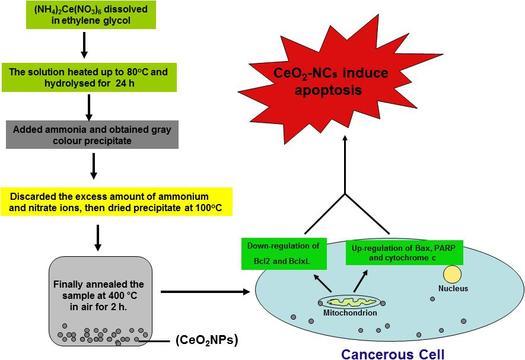

## 1. Introduction

Nanobiotechnology, a rapidly emerging field of advanced materials science, has expanded the possibility for advances in medical sciences such as gene-delivery systems, targeted drug delivery, and artificial implants [1–3]. Nanotechnology has been widely employed in applications such as cancer control, biomarker discovery, molecular biology, genetic engineering, cell engineering, drug delivery, skin care products, cosmetics, non-viral gene carriers, bio-analytical, clinical, diagnostics, and therapeutic fields [4,5]. The invention and development of nanomaterials represents one of the most potent and beneficial achievements that has advanced modern science and technology and allowed for the control of various conditions, including bacterial and fungal infections, as well as various types of cancer [6–8]. Cancer is considered to be one of the most insidious, complex, and heterogeneous diseases that occurs because of changes in the regulation of the proliferation of normal cells [9]. Colorectal cancer (CRC) represents a major cause of death in the USA. In 2016, approximately 49,190 individuals died as a consequence of CRC, while an estimated 95,270 people are diagnosed with the disease in USA [10] This type of cancer is very common in Western countries as one in 21 men and one in 23 women are predicted to develop the disease [11]. In this study, to explore the potential effects of cerium oxide nanocrystals (CeO_2_-NCs), we selected two CRC cell lines, HT29 and SW620 cells, for study. Despite advances in the diagnosis of cancer, the actual rate of treatment of this disease has yet to be markedly reduced. In CRC, normal cells progressively transform into malignant cells through initiation, promotion, and progression phases. Various studies have reported significant toxicity of nanomaterials against several types of cancer [12–14].

Recently, CeO_2_-NCs or nanoceria have been shown to exhibit a broad range of potential applications in medical science. For example, CeO_2_-NCs act as potential therapeutic agents against various diseases, including cancer, with the least toxicity towards normal cells and tissues [15,16]. CeO_2_-NCs have potential catalytic and antioxidant activities that resemble those of catalase and superoxide-dismutase and can act to protect cells from oxidative stress. However, several reports have demonstrated a protective role for CeO_2_-NCs towards oxidant-associated apoptosis [17], and many studies have confirmed that CeO_2_-NCs can induce oxidative stress *in vivo* and *in vitro* [18,19]. Furthermore, it was reported that CeO_2_-NCs can effectively reduce the proliferation of endothelial cells [20]. Therefore, CeO_2_-NCs could potentially be used to control the process of angiogenesis. The accumulation of reactive oxygen species (ROS) in cells is generally associated with adverse effects. ROS may promote the growth of tumors as a consequence of the deregulation of ROS scavengers and down-regulation of the expression of antioxidant enzymes [21]. It has been established that ROS are involved in changes in intracellular processes, so they represent a potential risk factor for cellular injury. It has also been shown that various types of NCs work as effective antioxidants and free radical scavengers. Thus, CeO_2_-NCs exhibit antioxidant properties that may counteract the progression of various diseases, including cancer, that are associated with oxygen levels and ROS generation [22]. Several studies that assessed the activity of CeO_2_-NCs in cancer showed that CeO_2_-NCs have anti-invasive effects against several types of cancer cells [23], and also confer radioprotection to normal cells and induce radio-sensitization [24] by controlling the extent of ROS and antioxidant enzyme activities [25]. Adverse health implications are rarely observed upon exposure to CeO_2_-NCs, and such outcomes differ between researchers, e.g. toxic effects on various organs such as the lung and kidney [26,27]. Recently, high cytotoxic effects of CeO_2_-NCs were reported in studies that used HT29 colorectal adenocarcinoma and 518A2 melanoma cell lines, while minimum cytotoxic effects were observed on other cell lines. Although present data on the medical applications of CeO_2_-NCs, including in cancer, are promising, conflicting reports exist regarding *in vitro* cytotoxicity. Moreover, data about the underlying mechanism and potential link to the induction of apoptosis are insufficient. Therefore, more research is required to confirm the mechanism of action of CeO_2_-NCs in apoptosis because of the possible cytotoxic effect of these compounds in human colorectal adenocarcinoma.

Herein, we present the chemical synthesis and characterization of CeO_2_-NCs and evaluate potential cytotoxic effects of CeO_2_-NCs on two human colon cancer cell lines. We also analyzed the potential effects of CeO_2_-NCs on the expression levels of Bcl2, BclxL, Bax, poly-ADP ribose-polymerase (PARP) and cytochrome c proteins, which all are involved in the apoptotic pathway. Furthermore, we also characterized the biocompatibility of CeO_2_-NCs on red blood cells (RBCs). Our findings illustrated that CeO_2_-NCs have sufficient cytotoxic potential against HT29 and SW620 cancer cell lines. We also observed that exposure of CeO_2_-NCs to the HT29 cancer cell line altered the normal expression levels of Bcl2, BclxL, Bax, PARP, and cytochrome c proteins.

## 2. Materials and methods

### 2.1. Materials

Ammonium cerium nitrate [(NH_4_)_2_Ce(NO_3_)_6_; 99.99%, BDH Chemicals, Poole, UK], C_2_H_5_OH, NH_4_OH, and ethylene glycol were utilized as starting chemicals without any additional purification steps. Nanopure water was used to prepare solutions. The advanced Millipore system (Milli-Q, Bedford, MA, USA) was used to generate ultrapure deionized water. All other materials used were of advanced reagent grade.

### 2.2. Synthesis and characterization of CeO_2_ nanocrystals

For the synthesis of CeO_2_-NCs, initially (NH_4_)_2_Ce(NO_3_)_6_ was solubilized in 50 ml ethylene glycol and then was warmed to 80°C. Subsequently, this solution was placed in a fresh 250 ml flask connected to a reflux condenser and hydrolyzed for ~24 h. The minimum quantity of ammonium hydroxide solution was mixed with the hydrolyzed solution and then was incubated to allow for precipitation. A grey colored precipitate was obtained, centrifuged, and washed three times with Milli-Q water to eliminate extra nitrate and ammonium ions before dehydration at 100°C. The ‘as prepared’ samples were annealed in air at 400°C for 2 h.

The synthesized product, CeO_2_-NCs (CeO_2_-NCs), was characterized using different spectroscopic and microscopic methods. X-ray diffraction (PANalytical X'Pert Pro, Netherlands) pattern equipped with Ni filter and Cu Kα (λ=1.54056 A) radiation was applied for phase identification. Furthermore, samples were prepared for electron microscopy (TEM) observations by fixing a droplet of a colloidal ethanol solution of the powder sample on a copper grid that was coated with carbon. The size and morphology of the test samples were inspected using a field emission transmission electron microscope (FETEM; JEOL, JEM-2100F, Tokyo, Japan) operating at an accelerating voltage of 200 kV. Excess solution was removed using fresh blotting paper. The TEM grid was permitted to air dry before sample analysis. Fourier transform infrared (FTIR) spectroscopy was performed to analyze the surface and chemical composition of CeO_2_-NCs. FTIR spectra was recorded from Vertex 80 (Bruker, USA) infrared spectrometer with KBr pellet technique.

UV-visible absorption spectra of CeO_2_-NCs were recorded with a Perkin-Elmer Lambda-40 spectrophotometer in the range 190–600 nm. Samples were placed in a 1 cm^3^ stoppered quartz cell with a 1 cm path length. FT-Raman spectroscopy was carried out to characterize the molecular structure of CeO_2_-NCs. Raman spectra were recorded using a Jobin Yvon Horiba HR800 UV Raman microscope (Wien, Austria) with a He–Ne laser that emitted at 632.8 nm. Photoluminescence spectra of CeO_2_-NCs were recorded at room temperature on a Horiba Synapse spectrometer equipped with a 1024 × 256 pixel CCD, in a detection range of 300 (efficiency: 30%) to 1000 nm (efficiency: 35%). For all measurements, a slit width of 100 microns was used, and the spectral resolution was confirmed to be better than 1 cm^−1^.

### 2.3. Cell culture in 75 cm^2^ tissue culture flasks

The human HT29 and SW620 colorectal cancer cell lines were seeded into 75 cm^2^ separate tissue culture flasks in 5 ml Dulbecco’s modified Eagle medium (DMEM). Culture medium contained 100 μg ml^–1^ streptomycin, 100 U ml^–1^ penicillin, 10% fetal bovine serum (FBS, Invitrogen, Carlsbad, CA, USA), 0.2% sodium bicarbonate, and 2 mmol l^–1^ L-glutamine. Cancer cell lines were cultured as adherent monolayers at 37°C with 5% CO_2_ in a humidified incubator. The trypan blue dye exclusion assay was performed to analyze cell viability prior to experiments and was carried out in accord with the manufacturer’s instructions. Batches of cells that exhibited more than 95% viability were used in experiments. Additionally, passages 8 and 10 for HT29 and SW620 cells were used in this study, respectively.

### 2.4. Cell viability assay for CeO_2_-NCS

The MTT assay was performed to analyze the anticancer activity of newly synthesized CeO_2_-NCs on HT29 and SW620 cells with a serial dilution of CeO_2_-NCs (5, 10, 20, 40, 80, 160, 320, and 640 μg ml^–1^). MTT assay results were based on the reduction of MTT reagent to a blue colored adduct by the mitochondrial dehydrogenase enzyme that is present in mitochondria of viable cells. Initially, HT29 and SW620 cells were harvested by trypsinization following the manufacturer’s recommended protocol. Subsequently, cells were cultured separately in 96-well plates with a density of ~1 × 10^6^ cells ml^–1^ and incubated at 37°C for 48 h in 5% CO_2_ using serial dilutions of CeO_2_-NCs (0, 5, 10, 20, 40, 80, 160, 320, and 640 μg ml^–1^) and various concentrations (0.5, 1.0, and 2.0 μM) of doxorubicin (positive control), as described in our previous studies [28,29]. Untreated HT29 and SW620 cells were used as a negative control. All experiments were carried out in triplicate. Freshly prepared 10 μl MTT reagent (5 mg ml^–1^ in phosphate-buffered saline, PBS) was transferred into each well after treatment with CeO_2_-NCs; control drug and plates were again incubated at 37°C for 4 h in an incubator with a 5% CO_2_ atmosphere. Finally, the resulting blue product (generated by the reduction of the tetrazolium salt of the MTT dye by mitochondrial dehydrogenase) was entirely dissolved in 100 μl dimethyl sulfoxide (DMSO). The absorbance of the resulting product (i.e. the optical density) was analyzed photometrically using a microplate reader at 595 nm. Absorbance from untreated HT29 and SW620 cells was used as a negative control. Cell viability was measured using the following formula, where OD stands for optical density.

$$\text{Cell viability}\;\left(\%\right)=\frac{\text{Mean OD of Samples}}{\text{Control OD}}\times 100$$

The average of three independent experiments was used to calculate results from each concentration to generate half maximal inhibitory concentration (IC_50_) values and estimate the surviving fraction of cells. Moreover, changes in the morphology of treated and untreated cells were analyzed by inverted microscopy.

### 2.5. Examination of morphological changes in HT29 cells by microscopy

Inverted microscopy was performed to examine changes in the morphology of treated HT29 cells and untreated control cells. Initially, HT29 cells were exposed to various concentrations of CeO_2_-NCs for 24 h. Untreated HT29 cells were used as a control group. Changes in the morphology of HT29 cells were examined using crystal violet staining. Additionally, 10% formalin was used to fix HT29 cells for 5 min. Formalin-fixed cells were stained with 0.2% crystal violet dye for 30–40 min. Cells were washed twice on slides using sterile distilled water. Images were captured at 20× using an inverted microscope with Microvisible software produced by Micros. Immunoblotting was used to observe potential changes in the levels of Bcl-2 and Bcl-xL (anti-apoptotic) and β-actin (control) proteins.

### 2.6. Mitochondrial membrane potential (MMP) assay

The protocol of Ahamed and Alhadlaq [30] was used to analyze MMP with few modifications. In brief, HT29 cells (5 × 10^5^ cells ml^–1^) were used in 20 mm plates. Cells were treated with 100 μg ml^–1^ CeO_2_-NCs for 18 h; untreated cells served as a control group. Cells were harvested and rinsed carefully with PBS. HT29 cells were exposed to 10 μg ml^–1^ of the fluorescent dye Rhodamine-123 (Rh-123) at 37°C for 30 min in a 5% CO_2_ incubator in the dark. Cells were rinsed again with PBS and then the fluorescence intensity of the Rh-123 dye was analyzed under a ZOE Fluorescent Cell Imager (Bio-Rad, Hercules, CA, USA). Images of treated and untreated (control) cells were captured.

### 2.7. Study of apoptotic pathways

The study of apoptotic pathways was performed by analyzing changes in the expression levels of different proteins along with those of β-actin in HT29 colorectal cancer cells.

#### 2.7.1. Estimation of changes in the expression of Bcl2 and BclxL proteins

The possible effect of CeO_2_-NCs on HT29 cells was measured by estimating changes in the expression levels of anti-apoptotic proteins (Bcl2, BclxL) and β-actin (control). Growing HT29 cells were exposed to 2.26 μg ml^–1^ CeO_2_-NCs for 24 h. Lysates of HT29 cells were cultured as described in our previous report [31]. Whole cell lysates were assayed to evaluate changes in the expression of soluble proteins using sodium dodecyl sulfate polyacrylamide gel electrophoresis (SDS-PAGE) immunoblotting. Blotted membranes were probed with antibodies against Bcl2 and BclxL (Santa Cruz Biotechnology), and β-actin (Sigma) to identify any possible changes in the expression level of anti-apoptotic proteins. The potential reactivity of antibodies against Bcl2, BclxL, and β-actin was observed using a horseradish peroxidase-conjugated secondary antibody (Santa-Cruz Biotechnology). Clarity western ECL substrate (Bio-Rad) was used to detect chemiluminescence with a C-Digit Blot Scanner (LI-COR Biosciences, Lincoln, NE, USA).

#### 2.7.2. Changes in the expression of PARP and cytochrome c proteins

Lysates were prepared from whole cells, as described in our previous report [31]. Solubilized proteins were detected by immunoblotting with anti-PARP (1:500; BioVision, San Francisco, CA, USA), anti-cytochrome c (1:200; Abcam, Cambridge, MA, USA), and anti-β-actin (1:10,000; Sigma, St Louis, MO, USA). Reactivity was estimated using a horseradish peroxidase-conjugated secondary antibody and Clarity western ECL substrate for chemiluminescence with a C-Digit Blot Scanner (LI-COR).

### 2.8. Biocompatibility assay for CeO_2_-NCs

A biocompatibility assay was performed to measure the percentage of hemolysis using 5 ml fresh blood. Initially, blood samples were obtained from a healthy volunteer in a sterile blood collection tube coated with EDTA. Each blood sample was collected with the consent of the healthy donor and according to ethical guidelines. Erythrocytes/RBCs were separated from whole blood by centrifugation for 10–15 min at 1500 rpm. The supernatant, which contained various plasma proteins and platelets, was removed. RBC pellets were rinsed carefully using sterile PBS. Subsequently, RBCs pellets (~2 ml) were mixed in 6 ml sterile PBS. Subsequently, 100 μl RBC suspension was added to 500 μl of the CeO_2_-NCs suspension (with an inhibitory concentration of 122.18 μg ml^–1^). The suspension of RBCs (100 μl) was carefully mixed in 500 μl PBS as a negative control and similarly 100 μl of the RBC suspension was mixed in sterile water (500 μl) as a positive control. All samples were vortexed briefly and incubated at 37°C in an incubator under static positions for 4 h. Next, all samples were vortexed carefully and centrifuged at 5500 rpm for 15 min. The upper aqueous portion was harvested without disturbing the lower layer and used to analyze the absorbance of hemoglobin at 575 nm using a BioTek Synergy HT Microplate Reader. All experiments were performed in triplicate. The percentage of hemolysis was analyzed based on the average of triplicates using the following formula [28]:

$$\%\;\text{of}\;\text{Hemolysis}=\;\frac{\left(\text{Sample}\text{absorbance}\;\text{-}\;\text{Absorbance}\;\;\text{of}\text{negative}\;\text{control}\;\right)}{\left(\text{Absorbance}\;\text{of}\text{positive}\;\;\text{control}\;\text{-}\;\text{Negative}\;\text{control}\right)}\times 100$$

### 2.9. Statistical analyses

All data presented in this article are means ± standard deviation (SD) of experiments performed in triplicate. The significance of differences between means was calculated using one-way analysis of variance (ANOVA). Significance was assigned at *p* < 0.05. All calculations were performed using the Prism software package (GraphPad Software, La Jolla, CA, USA).

## 3. Results and discussion

### 3.1. Crystallographic and morphological studies

The phase structural and crystalline properties of the prepared product were determined by XRD. Figure S1 shows a powder XRD pattern of sol-gel-prepared CeO_2_-NCs. XRD data showed that CeO_2_-NCs were properly crystallized and exhibited patterns that were in accord with a cubic structure. The main reflection peaks of CeO_2_ were analyzed for the diffraction pattern, and they corresponded to the (111), (200), (220) and (311) planes [32]. The observed reflection peaks could be indexed to the cubic fluorite structure of CeO_2_. No samples illustrated an extra peak that would correspond to any impurity phase of metal salt, which ruled out the formation of a secondary phase. The intensities and positions of the diffraction plane were well match with the JCPDS card No. (34-0394) and previously reported data [33]. The broadened peaks confirm the nanocrystalline nature of synthesized CeO_2_-NCs in the diffractogram. The robust peak (111) at 2θ = 28.4° and another peak (220) at 2θ = 47.17° were used to analyze the average crystallite size of CeO_2_-NCs, which according to the Scherrer equation were found to be ~10–15 nm.

A TEM micrograph of CeO_2_-NCs was also obtained (Figure S2). It could be observed that synthesized CeO_2_-NCs have an irregular shape and are slightly aggregated with a narrow size distribution. Similarly, Marques et al. [34] observed that ethylene glycol (EG) promoted the aggregation of nucleation seeds or tiny particles on the surface by potential interactions with the hydrogen bonding of H_2_O and with the polymer-OH groups. Additionally, the average particle size was reduced from 20 to 13 nm, which is consistent with the average particle size attained from the peak broadening in the XRD studies.

### 3.2. Optical properties

FTIR spectroscopy was performed to determine the surface chemistry of the synthesized CeO_2_-NCs, as shown in Figure S3. A strong absorption band between 400 and 500 cm^−1^ could represent an adsorption of metal oxygen (M-O) stretching band [35]. IR broad bands located at 1380, 1636, and 3450 cm^−1^ could be attributed to the νO-H symmetrical stretching and bending vibrations of physically absorbed H_2_O molecules on the NCs surface [35].

The KBr pellet technique was used to record FTIR spectra using a Perkin-Elmer 580B IR spectrometer in a range 400–4000 cm^−1^. Figure S4 shows the optical absorption spectrum of the NCs suspended in deionized water (suspensions were diffused in an ultrasonic bath before the absorption spectra were analyzed). An absorption band in the wavelength range of 250–450 nm was ascribed to a charge transfer transition from the O^2−^(2p) to Ce^4+^ (4f) orbital in CeO_2_. This finding indicates that the charge transfer transition of Ce^4+^ overlaps with the 4f^1^–5d^1^ transition of Ce^3+^. Raman-active phonon modes can be used to measure the structural order at a short range in the samples.

Figure S5 shows Raman spectra of the ‘as-synthesized’ CeO_2_-NCs in the range of 200–700 cm^−1^, which confirms the formation of a cubic fluorite phase. Unpolarized Raman scattering accumulated in the backscattering configuration with a 488 nm laser line at room temperature. The Raman active peak could be observed at 455 cm^−1^. The lattice expansion was used to elucidate systematic changes in the Raman peak with decreasing particle size, which coincides with the X-ray findings. The Raman spectrum also is in agreement with that of the pure CeO_2_-NCs, as previously reported in the literature [36]. The photoluminescence properties of CeO_2_-NCs were measured under 325 nm excitation wavelength using a Jobin Yvon Horiba spectrometer at room temperature, as shown in Figure S6. The CeO_2_-NCs exhibited a broad emission band within the 400–700 nm spectral range. It is well known that the emission band of ceria NCs resembles direct emission from the 2D(5d^1^) state to the two split 4f^1^ ground states of ^2^F_5/2_ and ^2^F_7/2_ that originate from spin orbit coupling.

### 3.3. Evaluation of the anticancer activity of CeO_2_-NCs by cytotoxicity assay

**Figure 1.**
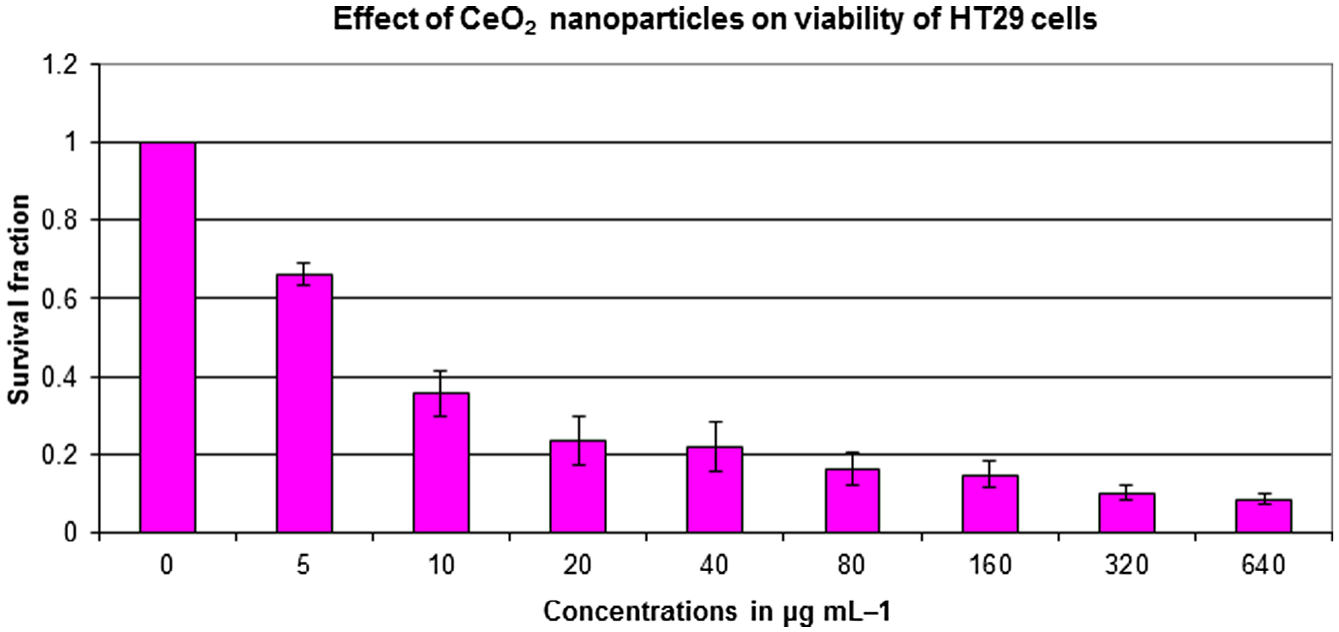
HT29 cell viability assessed based on the frequency of surviving cells after 48 h exposure to different concentrations of CeO_2_ nanoparticles by MTT assay. The IC_50_ was found to be 2.26 μg ml^–1^. Error bars indicate standard deviation (*n* = 3).

**Figure 2.**
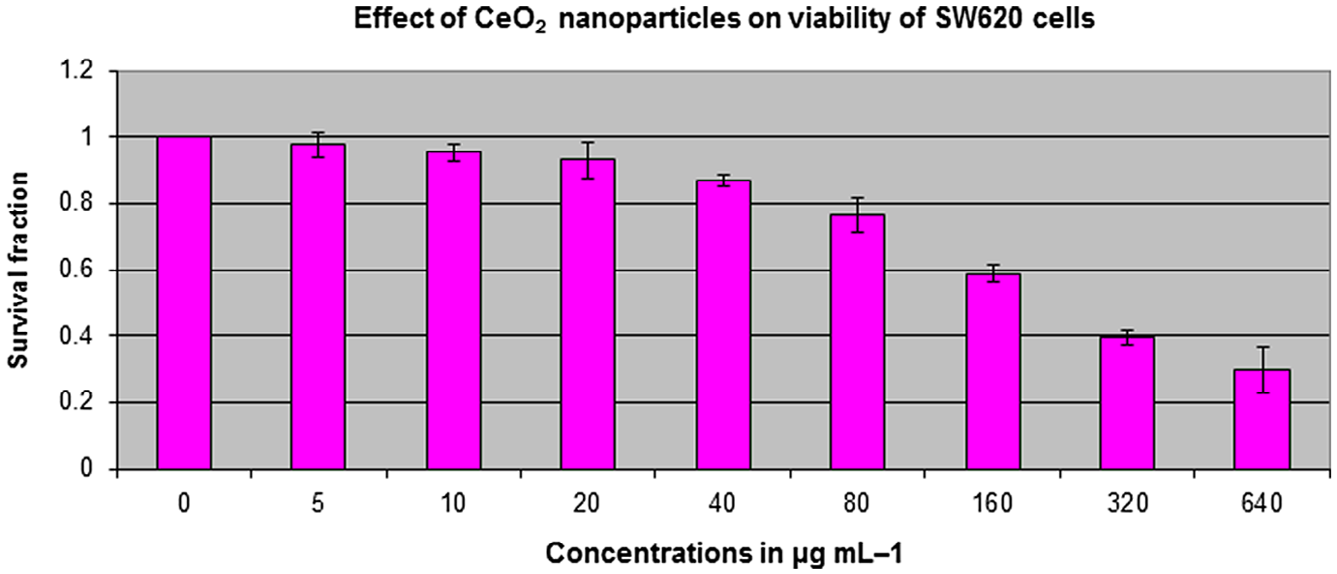
SW620 cell viability assessed based on the frequency of surviving cells after 48 h exposure to different concentrations of CeO_2_-NCs by MTT assay. The IC_50_ values were found to be 121.18 μg ml^–1^. Error bars indicate standard deviation (*n* = 3).

The potential antitumor activity of CeO_2_-NCs was assessed on HT29 and SW620 cell lines using serial dilutions of CeO_2_-NCs (5–640 μg ml^–1^) with the MTT assay. The CeO_2_-NCs were shown to effectively reduce the viability of HT29 and SW620 cells in a dose-dependent manner, as shown in Figures 1 and 2. The synthesized CeO_2_-NCs showed antitumor effects on HT29 and SW620 cells with a lower concentration of 50 μg ml^–1^. The viability of HT29 and SW620 cells decreased by 10% with 50 μg ml^–1^ CeO_2_-NCs, while at 100 μg ml^–1^ the viability of HT29 and SW620 cells was remarkably reduced up to 46%. The mechanism of action of CeO_2_-NCs remains the subject of some debate. We used an aqueous environment to prepare CeO_2_-NCs, which has large amount of hydroxyl groups on the surface of nanocrystals. Because of these surface hydroxyl groups, CeO_2_-NCs are biocompatible and less toxic. Moreover, they may be considered to be eco-friendly and do not pose significant environmental hazards, in contrast to those compounds used for the chemical reduction method [37]. The cytotoxic effects of CeO_2_-NCs on colon cancer cell lines suggest that CeO_2_-NCs can play an important role in the development of drugs against colorectal cancer. Additionally, the results of the MTT assay with various concentrations of positive control doxorubicin (0.5, 1.0, and 2.0 μM), as shown in our recent report, indicated that the control drug had considerable effects on HT29 cells [28].

The present study explored the cytotoxic potential of CeO_2_-NCs on the HT29 and SW620 human colorectal cancer cell lines. CeO_2_-NCs showed excellent anticancer activity with half maximal inhibitory concentration (IC_50_) value of 2.26 and 121.18 μg ml^–1^ against HT29 and SW620 cells, respectively. Our data were in agreement with previously published reports that cell lines required high drug concentrations and/or more incubation times than HT29 cells to achieve cytotoxic effects [28]. It had been previously observed that cytotoxicity of different NPs in different cancerous cell lines depends upon the concentration of NPs used and the exposure time [38]. Previous studies reported the non-significant toxicity of CeO_2_-NCs against different normal epithelial cell lines [23].

### 3.4. Examination of morphological changes in HT29 cells by microscopy

**Figure 3.**
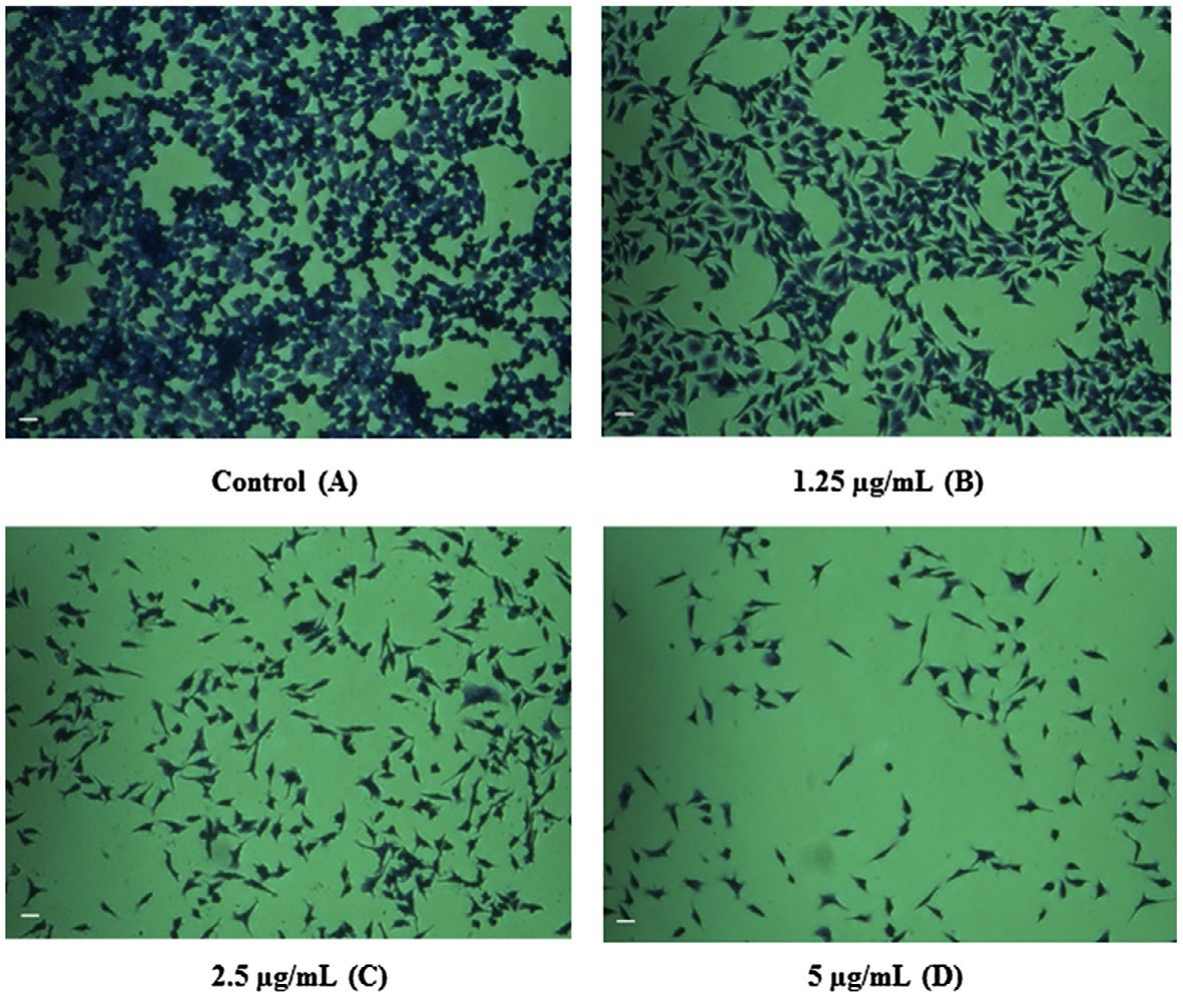
Inverted microscopy results show the potential effect of CeO_2_-NCs by crystal violet staining. **(A)** No significant effects were observed on untreated HT29 cells (control). **(B–D)** HT29 cells were treated with different concentrations of CeO_2_-NCs. Alterations in morphology and reductions in cell number indicate the activity of CeO_2_-NCs. Bars = 10 μm.

The outcomes of invert microscopy using the HT29 cell line demonstrated a reduction in the numbers of cells in response to various concentrations of CeO_2_-NCs. The control group of untreated cells showed no change in the number of cells or in cell morphology compared with CeO_2_-NCs-treated cells by crystal violet staining (Figure 3A–D). Treated cells exhibited morphological alterations (i.e. they became rounded), which is a possible sign of apoptosis. Moreover, inhibition of cell growth, loss of membrane integrity, and condensation of the cytoplasm could be detected in HT29 cells treated with CeO_2_-NCs when compared with untreated controls. These findings indicate that CeO_2_-NCs induce cell death by inhibiting the proliferation of HT29 cells.

### 3.5. MMP assay

**Figure 4.**
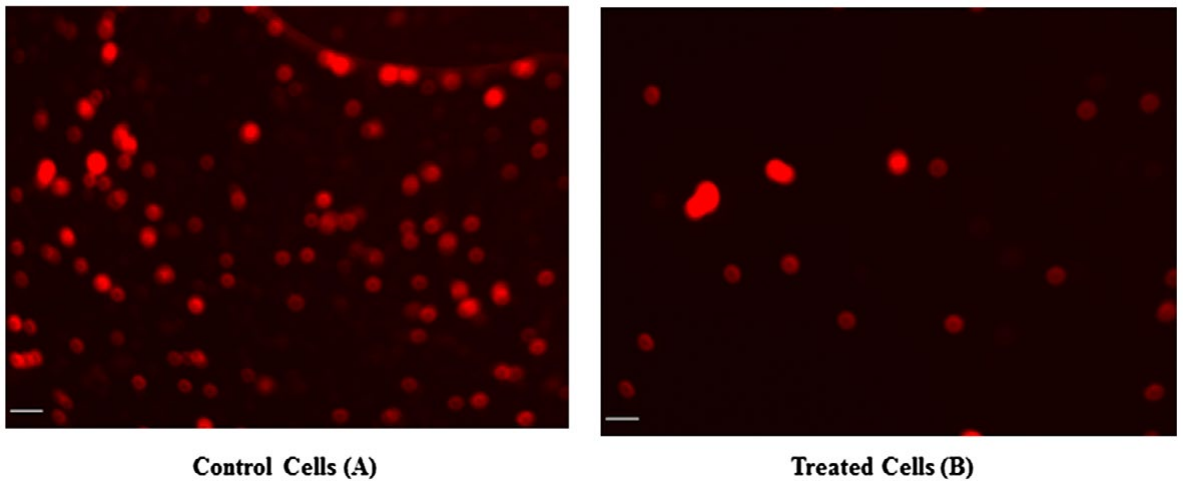
The microphotographs obtained by fluorescence microscopy indicate changes in mitochondrial membrane potential (MMP). **(A)** Untreated HT29 cells were used as a control. **(B)** HT29 cells showed significant changes in mitochondrial membrane potential (MMP) after treatment with CeO_2_-NCs. Bars = 10 μm.

It is well recognized that the MMP of cells is reduced during apoptosis. Potential differences in the MMP of CeO_2_-NCs-treated and -untreated HT29 cells were analyzed by measuring the intensity of fluorescence using a mitochondria-specific Rh-123 dye. The MMP assay results revealed that CeO_2_-NCs reduced the MMP in HT29 cells when compared with untreated cells (Figure 4A–B). These MMP assay results supported the crystal violet-staining findings and demonstrated the cytotoxic potential of CeO_2_-NCs.

### 3.6. Study of the apoptotic pathway

CeO_2_-NCs have been used in different therapeutic and industrial applications. In the present study, a molecular approach was used to dissect the apoptotic pathway linked to cell death by estimating changes in the expression of anti-apoptotic (Bcl2 and BclxL) and pro-apoptotic (Bax, PARP and cytochrome c) proteins, along with control proteins. These studies were important because limited data are available about the molecular mechanism activated by CeO_2_-Ns that affects the protein expression levels of pro- and anti-apoptotic genes.

#### 3.6.1. Estimation of changes in the expression of Bcl2 and BclxL proteins

**Figure 5.**
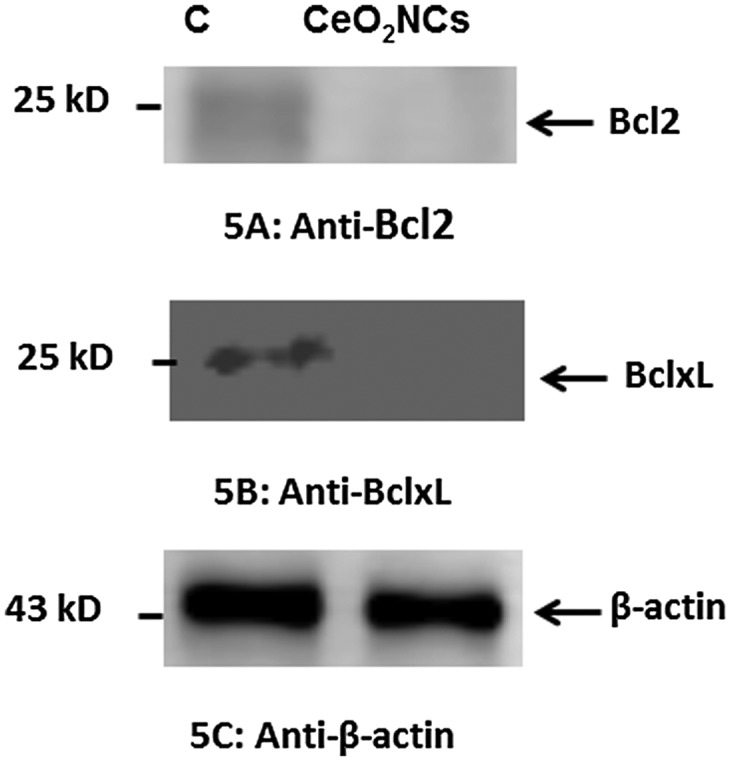
Western blot assessment of the expression of the anti-apoptotic proteins Bcl-2, Bcl-xL, and β-actin (control) in HT29 cells treated with 2.26 μg ml^–1^ CeO_2_ NCs for 24 h; controls were untreated HT29 cells. Immunoblot images show the expression levels of anti-apoptotic proteins. (A) Down-regulation of Bcl-2 expression was observed by immunoblot. (B) Down-regulation of Bcl-xL expression was observed by immunoblot. (C) Expression levels of β-actin (control) were unchanged.

Immunoblotting was carried out to examine possible changes in the expression levels of Bcl2 and BclxL proteins in HT29 cells. The expression levels of selected genes were compared with those of a housekeeping gene, β-actin, that served as a control. HT29 cells were exposed to synthesized CeO_2_-NCs at a concentration of 2.26 μg ml^–1^ for 24 h. CeO_2_-NCs induce cell death, as indicated by the down-regulation of Bcl2 family anti-apoptotic proteins. These experiments indicated that CeO_2_-NCs inhibited the expression of Bcl2 and BclxL when compared with β-actin and the untreated negative control (Figure 5A–B). No significant changes compared with the expression of β-actin were observed (Figure 5C).

Several reports have shown that down-regulation of the expression of Bcl2 and BclxL increased apoptotic potential [39,40]. By contrast, the up-regulation of Bcl2 and BclxL expression inhibits apoptosis [39]. Our present findings indicated that CeO_2_-NCs markedly reduced cell proliferation by inducing the down-regulation of Bcl2 and BclxL expression. Therefore, the immunoblot findings were in accord with those of the MTT assay.

#### 3.6.2. Estimation of changes in expression of PARP and cytochrome c proteins

**Figure 6.**
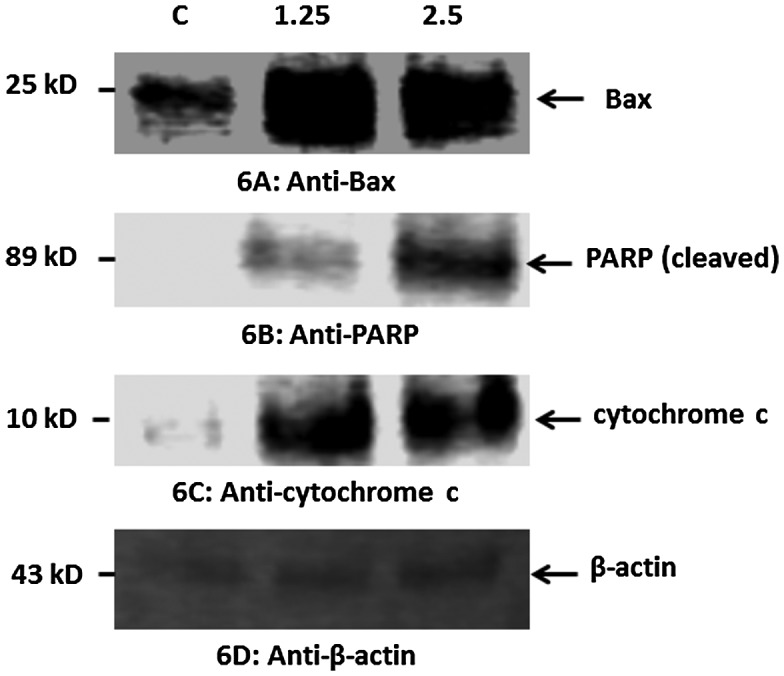
Western blot analysis of the pro-apoptotic proteins Bax, PARP, cytochrome c, and β-actin (control) in HT29 cells treated with 2.26 μg ml^–1^ CeO_2_-NCs for 24 h; untreated HT29 cells served as controls. Immunoblot analyses of the expression levels of apoptotic proteins are shown. (A). Up-regulation of the expression of Bax. (B). Up-regulation of the expression of PARP. (C). Up-regulation of the expression of cytochrome c. (D) No significant change in the expression of β-actin (control).

Our data indicated that treatment of HT29 cells with CeO_2_-NCs induced the expression of Bax, a pro-apoptotic protein (Figure 6A). Furthermore, CeO_2_-NCs could also induce PARP cleavage in HT29 cells (Figure 6B). PARP is a protein that is involved in various cellular processes, such as DNA repair and programmed cell death. PARP can be activated during the DNA damage and cellular stress responses. Caspase activation results in PARP cleavage during apoptosis. Our results demonstrated that cytochrome c was markedly increased in HT29 cells exposed to CeO_2_-NCs when compared to controls (Figure 6C–D). Cytochrome c released from mitochondria promotes the induction of apoptosis. These findings indicate that CeO_2_-NCs remarkably inhibit cell growth by inducing apoptotic pathways.

### 3.7. Biocompatibility assay of CeO_2_-NCs

**Figure 7.**
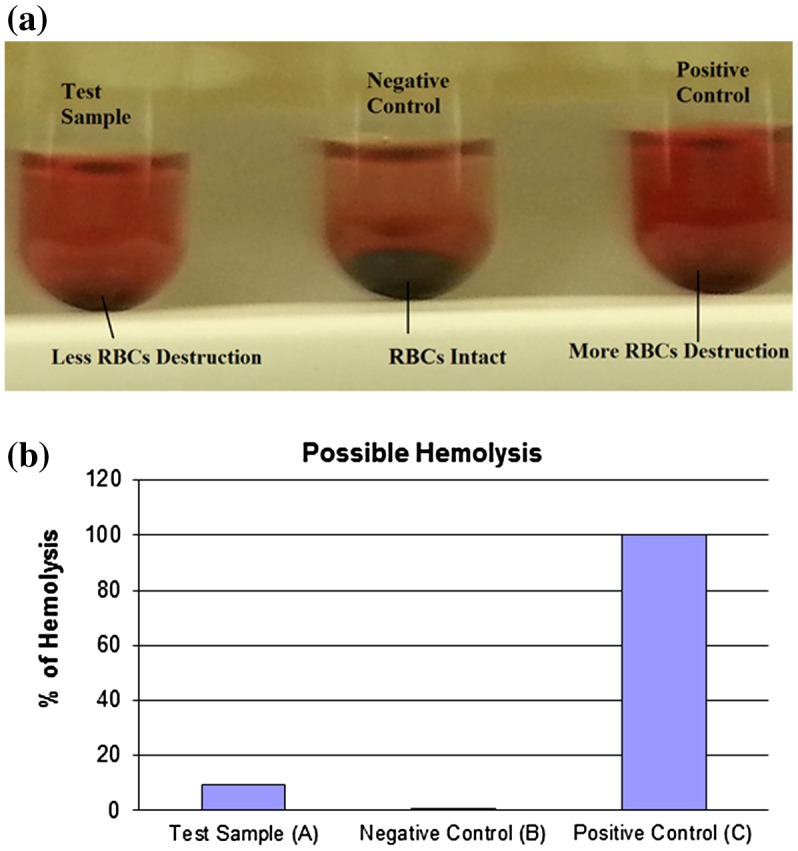
An *in vitro* biocompatibility assay shows possible hemolysis of CeO_2_ NCs compared with both positive and negative controls. **(A)** The test sample (CeO_2_-NCs) showed minimum destruction of RBCs, and the negative sample exhibited no RBC destruction compared with the positive control. **(B)** The percentage of hemolysis in test samples and the positive control.

*In vitro* blood hemolysis was measured using a biocompatibility assay. The assay was used to analyze hemolysis considering the toxic nature of synthesized CeO_2_-NCs, as described in a recent study [40]. Hemolysis may act as a potential cause of anemia in various pathological settings [28,41]. Human RBCs showed the hemolytic potential of CeO_2_-NCs (Figure 7A). These findings suggest that the hemolytic potential of CeO_2_-NCs was less significant compared with that of a positive control, which implies that less damage occurs after the use of CeO_2_-NCs. Our *in vitro* experiments demonstrated the biocompatibility of the synthesis of CeO_2_-NCs, which indicated their suitability and biocompatibility for future drug development and applications in the field of biomedical science. As shown in the graphs, hemolysis was less frequent in the test sample when compared with the positive control (Figure 7B). Therefore, the minimum hemolysis associated with CeO_2_-NCs suggests the better suitability and biocompatibility of synthesized NCs for applications in biomedical science, such as the treatment and management of different diseases, including cancer.

## 4. Conclusions

CeO_2_-NCs were successfully synthesized and their physical properties were optimized using various techniques. The FETEM micrograph of prepared CeO_2_ NCs showed that the particles were well dispersed with a diameter range of 13–20 nm, which was confirmed by XRD. An *in vitro* cell cytotoxicity assay showed that CeO_2_-NCs had cytotoxic potential. It is essential to determine whether other molecular mechanisms in different pathways are involved. The apoptotic pathway assessments with Western blot assays confirmed that CeO_2_-NCs targeted to HT29 cancer cell lines altered the expression levels of the anti-apoptotic proteins Bcl2 and BclxL, and increased the expression of the Bax, PARP, and cytochrome c proteins. Bcl2 protein can exert anti-apoptotic effects, while the Bax protein is potentially involved in the regulation of pro-apoptotic activities [42]. Certain ratios of Bax/Bcl-2 protein are associated with cell death, which determines the death or life of particular cells in response to an apoptotic stimulus; a higher ratio of Bax/Bcl2 robustly inhibits cellular resistance to apoptotic stimuli.

Furthermore, the reduced effect of CeO_2_-NCs on RBCs in the hemolysis assay suggests biocompatibility. Therefore, the synthesis of CeO_2_-NCs in the present study may be further examined for use in the preclinical development of novel anti-cancer drugs, which could improve the health of humans because of the significant anticancer potential of such compounds. Studies of the anti-cancer properties of CeO_2_-NCs are in an advanced stage for the development of next-generation drugs for *in vivo* trials.

## Conflict of interest

The authors have declared that they have no potential conflict of interest related to this study.

## Supplemental data

The supplemental material for this paper is available at https://doi.org/10.1080/14686996.2017.1319731.

## Supplementary Material

Electronic_Supplementary_Material.doc

## References

[CIT0001] Curtis A, Wilkinson C (2001). Nantotechniques and approaches in biotechnology. Trends Biotechnol.

[CIT0002] Chan WC, Nie S (1998). Quantum dot bioconjugates for ultrasensitive nonisotopic detection. Science.

[CIT0003] Langer R (2001). Drug delivery. Drugs on target. Science.

[CIT0004] De Jong WH, Borm PJ (2008). Drug delivery and nanoparticles:applications and hazards. Int J Nanomed.

[CIT0005] He Y, Chen D, Li M (2014). Rolling circle amplification combined with gold nanoparticles-tag for ultra sensitive and specific quantification of DNA by inductively coupled plasma mass spectrometry. Biosens Bioelectron.

[CIT0006] Wesselinova D (2011). Current major cancer targets for nanoparticle systems. Curr Cancer Drug Targets.

[CIT0007] Liu L, Xu K, Wang H (2009). Self-assembled cationic peptide nanoparticles as an efficient antimicrobial agent. Nat Nanotechnol.

[CIT0008] Yu L, Zhang Y, Zhang B (2014). Enhanced antibacterial activity of silver nanoparticles/halloysite nanotubes/graphene nanocomposites with sandwich-like structure. Sci Rep.

[CIT0009] Arnold M, Pandeya N, Byrnes G (2015). Global burden of cancer attributable to high body-mass index in 2012: a population-based study. Lancet Oncol.

[CIT0010] Siegel RL, Miller KD, Jemal A (2016). Cancer statistics, 2016. CA Cancer J Clin.

[CIT0011] Lynch HT, de la Chapelle A (2003). Hereditary colorectal cancer. N Engl J Med.

[CIT0012] McCarroll J, Teo J, Boyer C (2014). Potential applications of nanotechnology for the diagnosis and treatment of pancreatic cancer. Front Physiol.

[CIT0013] Drbohlavova J, Chomoucka J, Adam V (2013). Nanocarriers for anticancer drugs–new trends in nanomedicine. Curr Drug Metab.

[CIT0014] Garbuzenko OB, Mainelis G, Taratula O (2014). Inhalation treatment of lung cancer: the influence of composition, size and shape of nanocarriers on their lung accumulation and retention. Cancer Biol Med.

[CIT0015] Wason MS, Zhao J (2013). Cerium oxide nanoparticles: potential applications for cancer and other diseases. Am J Transl Res.

[CIT0016] Gao Y, Chen K, Ma JL (2014). Cerium oxide nanoparticles in cancer. Onco Targets Ther.

[CIT0017] Celardo I, De Nicola M, Mandoli C (2011). Ce(3)+ ions determine redox-dependent anti-apoptotic effect of cerium oxide nanoparticles. ACS Nano.

[CIT0018] Park EJ, Choi J, Park YK (2008). Oxidative stress induced by cerium oxide nanoparticles in cultured BEAS-2B cells. Toxicology.

[CIT0019] Mittal S, Pandey AK (2014). Cerium oxide nanoparticles induced toxicity in human lung cells: role of ROS mediated DNA damage and apoptosis. Biomed Res Int.

[CIT0020] Lord MS, Tsoi B, Gunawan C (2013). Anti-angiogenic activity of heparin functionalised cerium oxide nanoparticles. Biomaterials.

[CIT0021] Waris G, Ahsan H (2006). Reactive oxygen species: role in the development of cancer and various chronic conditions. J Carcinog.

[CIT0022] Elswaifi SF, Palmieri JR, Hockey KS (2009). Antioxidant nanoparticles for control of infectious disease. Infect Disord Drug Targets.

[CIT0023] Pesic M, Podolski-Renic A, Stojkovic S (2015). Anti-cancer effects of cerium oxide nanoparticles and its intracellular redox activity. Chem Biol Interact.

[CIT0024] Wason MS, Colon J, Das S (2013). Sensitization of pancreatic cancer cells to radiation by cerium oxide nanoparticle-induced ROS production. Nanomedicine.

[CIT0025] Colon J, Hsieh N, Ferguson A (2010). Cerium oxide nanoparticles protect gastrointestinal epithelium from radiation-induced damage by reduction of reactive oxygen species and upregulation of superoxide dismutase 2. Nanomedicine.

[CIT0026] Aalapati S, Ganapathy S, Manapuram S (2014). Toxicity and bio-accumulation of inhaled cerium oxide nanoparticles in CD1 mice. Nanotoxicology.

[CIT0027] Fall M, Guerbet M, Park B (2007). Evaluation of cerium oxide and cerium oxide based fuel additive safety on organotypic cultures of lung slice. Nanotoxicology.

[CIT0028] Khan S, Ansari AA, Khan AA (2016). Design, synthesis and *in vitro* evaluation of anticancer and antibacterial potential of surface modified Tb(OH)3@SiO2 core–shell nanoparticles. RSC Adv.

[CIT0029] Khan S, Ansari AA, Khan AA (2017). In vitro evaluation of cytotoxicity, possible alteration of apoptotic regulatory proteins, and antibacterial activity of synthesized copper oxide nanoparticles. Colloids Surf B Biointerfaces.

[CIT0030] Ahamed M, Alhadlaq HA (2014). Nickel nanoparticle-induced dose-dependent cyto-genotoxicity in human breast carcinoma MCF-7 cells. Onco Targets Ther.

[CIT0031] Ahmad R, Raina D, Trivedi V (2007). MUC1 oncoprotein activates the IkappaB kinase beta complex and constitutive NF-kappaB signalling. Nat Cell Biol.

[CIT0032] Ansari AA, Sumana G, Pandey MK (2009). Sol–gel-derived titanium oxide–cerium oxide biocompatible nanocomposite film for urea sensor. J Mater Res.

[CIT0033] Khan S, Ansari AA, Khan AA (2015). In vitro evaluation of anticancer and antibacterial activities of cobalt oxide nanoparticles. J Biol Inorg Chem.

[CIT0034] Marques VS, Cavalcante LS, Sczancoski JC (2010). Effect of different solvent ratios (water/ethylene glycol) on the growth process of CaMoO4 crystals and their optical properties. Cryst Growth Des.

[CIT0035] Ansari AA, Solanki PR, Malhotra BD (2009). Hydrogen peroxide sensor based on horseradish peroxidase immobilized nanostructured cerium oxide film. J Biotechnol.

[CIT0036] Zhang F, Chan SW, Spanier JE (2002). Cerium oxide nanoparticles: Size-selective formation and structure analysis. Appl Phys Lett.

[CIT0037] Chen L, McCrate JM, Lee JC (2011). The role of surface charge on the uptake and biocompatibility of hydroxyapatite nanoparticles with osteoblast cells. Nanotechnology.

[CIT0038] Lu X, Qian J, Zhou H (2011). In vitro cytotoxicity and induction of apoptosis by silica nanoparticles in human HepG2 hepatoma cells. Int J Nanomed.

[CIT0039] Figueroa B, Chen S, Oyler GA (2004). Aven and Bcl-xL enhance protection against apoptosis for mammalian cells exposed to various culture conditions. Biotechnol Bioeng.

[CIT0040] Khan S, Ansari AA, Khan AA (2016). In vitro evaluation of anticancer and biological activities of synthesized manganese oxide nanoparticles. Med Chem Commun.

[CIT0041] Sathishkumar G, Bharti R, Jha PK (2015). Dietary flavone chrysin (5,7-dihydroxyflavone ChR) functionalized highly-stable metal nanoformulations for improved anticancer applications. RSC Adv.

[CIT0042] Reed JC (2006). Proapoptotic multidomain Bcl-2/Bax-family proteins: mechanisms, physiological roles, and therapeutic opportunities. Cell Death Differ.

