# Supplementary material for: Evaluation of in vitro cytotoxicity, biocompatibility, and changes in the expression of apoptosis regulatory proteins induced by cerium oxide nanocrystals
Source: Sci Technol Adv Mater. 2017 May 31;18(1):364–73. doi: 10.1080/14686996.2017.1319731 (PMC5468938; doi:10.1080/14686996.2017.1319731)
Supplement: Electronic_Supplementary_Material.doc [file tsta_a_1319731_sm4050.doc]

## Evaluation of in vitro cytotoxicity, biocompatibility, and changes in the expression of apoptosis regulatory proteins induced by cerium oxide nanocrystals

**Shahanavaj Khan^ab^*, Anees A. Ansari^b^, Christian Rolfo^c^, Andreia Coelho^c^, Maha Abdulla^d^, Khayal Al-Khayal^d^, and Rehan Ahmad^d^**

*^a^Nanomedicine & Biotechnology Research Unit, Department of Pharmaceutics, College of Pharmacy, PO Box 2457, King Saud University, Riyadh 11451, Saudi Arabia*

*^b^Department of Bioscience, Shri Ram Group of College (SRGC), Muzaffarnagar, UP, India*

*^c^King Abdullah Institute for Nanotechnology, King Saud University, P.O. Box: 2455, Riyadh 11451, Saudi Arabia*

*^d^Phase I- Early Clinical Trials Unit, Oncology Department and Multidisciplinary Oncology Center Antwerp (MOCA) Antwerp University Hospital, Edegem, Belgium*

*^e^Colorectal Research Center, College of Medicine King Saud University, P.O. Box: 2455, Riyadh 11451, Saudi Arabia*

***Corresponding author:** Dr. Shahanavaj Khan

**E-mail:** khan.shahanavaj@gmail.com

**Figure S1.** X-ray diffraction pattern of CeO_2_ NCs.

**Figure**

**Figure S2.** Typical FE-TEM micrograph of the synthesized CeO_2_ NCs.


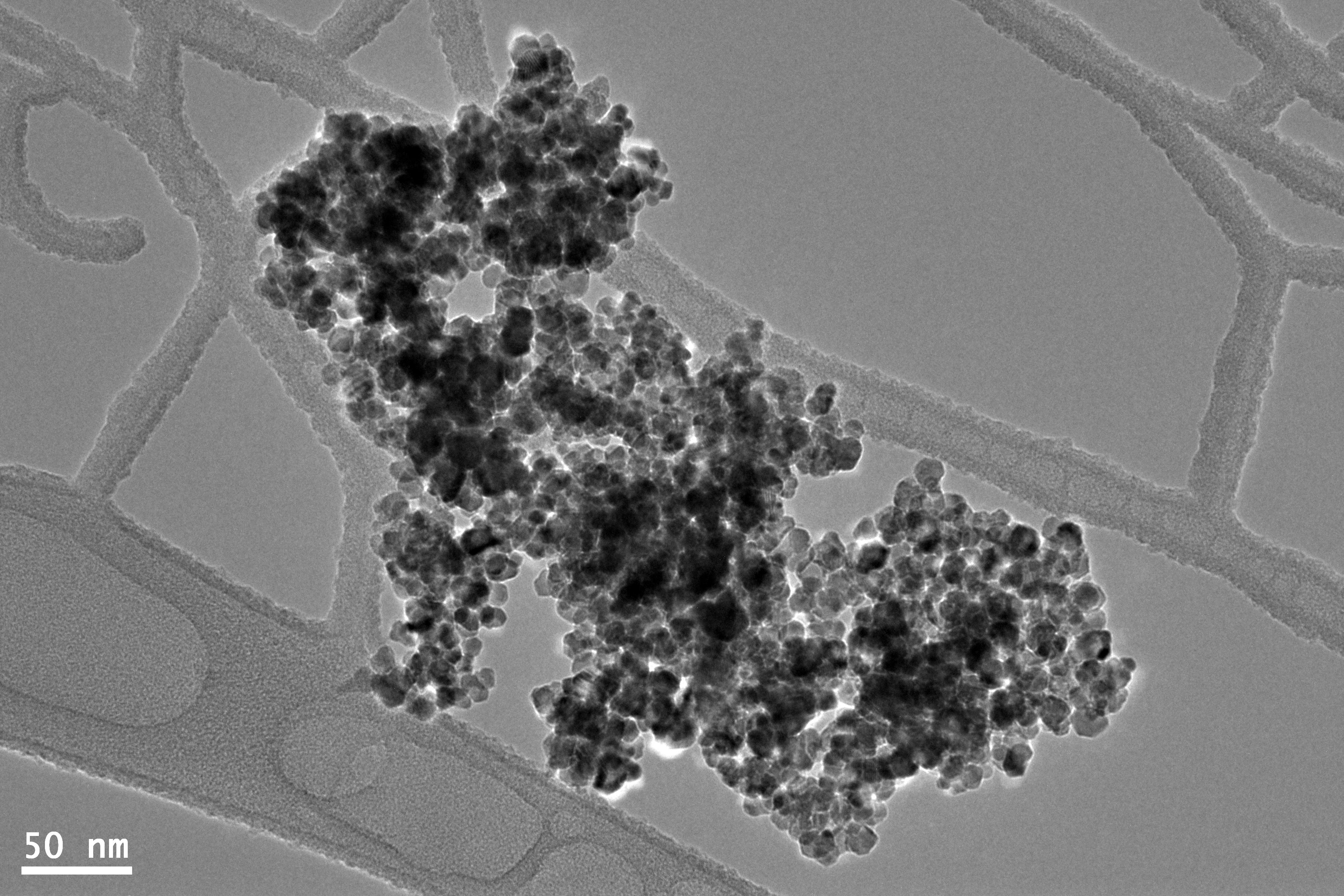


**Figure S3.** FTIR spectrum of the of CeO_2_ NCs.

**Figure S4.** UV-vis absorption spectrum of the of CeO_2_ NCs suspended in dionized water.

**Figure S5.** FT-Raman spectrum of the of CeO_2_ NCs

**Figure S6.** Photoluminescence spectrum spectrum of the of CeO_2_ NCs
